# Seroprevalence of brucellosis among animal handlers in West Bengal, India: an occupational health study

**DOI:** 10.3934/microbiol.2024001

**Published:** 2024-01-02

**Authors:** Dolanchampa Modak, Silpak Biswas, Agnibho Mondal, Malabika Biswas, Maria Teresa Mascellino, Banya Chakraborty, Simmi Tiwari, Ajit Dadaji Shewale, Tushar Nale, Rupali Dey

**Affiliations:** 1 Department of Microbiology, School of Tropical Medicine, Kolkata 700073, India; 2 Department of Public Health and Infectious Diseases, Sapienza University of Rome, Rome 00185, Italy; 3 Centre for One Health, National Centre for Disease Control (NCDC), Ministry of Health & Family Welfare, Delhi 110054, India

**Keywords:** Zoonotic disease, brucellosis, animal handlers, seroprevalence

## Abstract

Brucellosis is a highly contagious zoonotic disease and a major human health problem worldwide. Due to its ways of transmission, direct or indirect contact with infected animals or their contaminated biological products, the disease exhibits strong occupational association with animal handlers comprising a significant population at risk. This study was undertaken to estimate the seroprevalence of brucellosis in animal handlers and to understand the epidemiological and serological aspects of the same. The animal handlers from the state of West Bengal, India were included in this study. It was a prospective and observational cohort study from November 2021 to March 2022. A total of 669 sera samples were collected from animal handlers and tested using various serological tests for *Brucella* antibodies. All serum samples were tested using the Rose Bengal plate test (RBPT), standard tube agglutination test (STAT), and enzyme-linked immunosorbent assay (ELISA). 106 (15.8%) patients were diagnosed with brucellosis among the total number of patients tested. Most of the patients affected with brucellosis belonged to the age group 51–60 years (23.5%). The seropositivity rate in male animal handlers was higher than female animal handlers in this study. More studies are needed to understand the occupational association of this disease. Awareness programs, safe livestock practices, and prevention of the disease by timely diagnosis must be implemented in order to control human brucellosis.

## 1. Introduction

Brucellosis is one of the most common zoonotic diseases worldwide. Brucellosis is caused by a Gram-negative coccobacilli belonging to the genus *Brucella* (family Brucellaceae) [1]–[5]. Among the prevalent species, *Brucella melitensis, Brucella abortus, Brucella suis* and *Brucella canis* are pathogenic to humans [6],[7]. In humans, the disease is characterized by a variety of manifestations including fever, night sweats, myalgia, arthralgia, and weakness [8]. The wide spectrum of clinical manifestations and the lack of pathognomonic symptoms make human brucellosis difficult to clinically diagnose and distinguish from several febrile conditions that often occur in the same areas. Therefore, laboratory tests are essential for diagnosing the disease. Brucellosis is associated with loss of livestock productivity and trade, thus incurring massive economic losses [9].

In spite of successful eradication attempts in many countries around the world, brucellosis exists as a potent animal and human health issue in developing as well as in developed countries [10]. In developed countries, it is prevalent in wild animals and can be a threat due to spill-over infection potential. Brucellosis is considered an endemic disease in India [9]. Many Indian people have close contact with domestic animals because of their occupation, particularly those involved in agriculture. Therefore, they have an increased risk of contracting many zoonotic diseases including brucellosis [11]. A study by Shukla et al. [12] in different Indian states showed that the overall seroprevalence of brucellosis from tertiary care health settings was 11% (772/7026). The majority of positive cases were from the states of Madhya Pradesh (58.1%), Maharashtra (38.8%) and Chhattisgarh (2.9%). Adults and females were more vulnerable among the study population [12]. Dutta et al. [13] investigated the presence of childhood brucellosis cases in Eastern zone of India. The findings from this study revealed the higher percentage of infection in female children (14.3%) than in male children (10.9%) [13]. Seroprevalence of 8.5% was reported in dairy workers by Mathur in their older study [14]. Looking at studies over time (from 1986 to 2011) there was wide variation of the prevalence of human brucellosis in India, such as 0.8% in Kashmir, 6.8 % in Varanasi, 8.5% in Gujarat, 11.51 % in Andhra Pradesh, 19.83% in Maharashtra, and 26.6 % in Ludhiana [15]–[20]. It is estimated that less than 10% of cases of human brucellosis are recognized and treated in India [21]. A recent rise of the disease in this country has been attributed to intensified developments in the dairy industry resulting in increased livestock population [9]. A high seroprevalence of anti-*Brucella* antibodies has been noted among veterinarians and veterinary pharmacists [22] in previous studies. Along with veterinary professionals, animal handlers comprise a significant population at risk of contracting brucellosis due to their continued involvement in health and management of livestock.

In India, the veterinary services fall under the purview of the state government. Most of the states have three types of veterinary health care workers: (1) qualified registered veterinarians, (2) paraveterinarians, and (3) animal handlers [23]. The animal handlers are engaged in artificial insemination, vaccination, and deworming of cattle [24] as part of veterinary services. They are consequently exposed to all possible routes of transmission of *Brucella* spp. including contact with secretions of diseased animals and needle stick injuries while vaccinating female calves. Handling of potentially infected animals, contaminated biological materials, and live attenuated anti-brucellosis vaccines are risk factors for human brucellosis. However, more detailed knowledge about particular risk factors to each occupation, as well as the measurement of these risks is still scarce. In fact, there is a need for more accurate data on the epidemiology of job-related brucellosis to allow the implementation of more effective preventive measures, which will reduce the impact of the disease in groups exposed by their work activities.

There are presently three live *Brucella* vaccines available commercially: *B. abortus* strain 19 (S19), *B. abortus* strain RB51 (RB51), and *B. melitensis* strain Rev 1 (Rev1) in animals [25]. Among these vaccines, S19 is most commonly used in all vaccination programs in India. It is a modified live culture vaccine [25]. However, *Brucella* vaccines have been documented to cause human brucellosis if accidental exposure occurs [26].

In India there exists a dearth of studies documenting brucellosis in animal handlers associated with vaccination program. Therefore, the objective of the present study was to (1) estimate the prevalence of brucellosis in animal handlers accidently exposed to S19 vaccine and (2) understand the epidemiological and serological aspect of the same. The serodiagnosis of brucellosis is mostly based on consensual criteria such as given titer in agglutination assay, a cut-off ELISA reading value, etc. Furthermore, the sensitivity and specificity of any serological test for brucellosis depends highly among other factors, on local epidemiological conditions [27]. The results of serological tests for brucellosis require interpretation that is often difficult and inconclusive [28]. Therefore, at least two positives out of three serological tests were used as criteria for diagnosis of brucellosis in surveillance in this study [29].

## 2. Materials and methods

### 2.1. Study design and study population

It was a prospective and observational cohort study conducted by the School of Tropical Medicine, Kolkata, India from November 2021–March 2022. The study was done by the Department of Microbiology and Department of Tropical Medicine and was supported by National One Health Program for Prevention and Control of Zoonoses (NOHP-PCZ), National Centre for Disease Control (NCDC), Delhi, India. The target population included animal handlers from the state of West Bengal, India, with a history of accidental exposure to *Brucella abortus* vaccine (S19 strain). An accidental exposure was defined as a needle stick injection through the skin or sprays or splash into the eye or broken skin of a human while handling the S19 vaccine [30].

### 2.2. Sample collection

Blood samples (2 mL) were collected from animal handlers reporting to the Outpatient Department of the School of Tropical Medicine (Kolkata) from various districts of West Bengal. Information regarding age, sex, geographic location, type of animal handling activity, history of exposure, clinical history, and other relevant details was obtained after seeking consent from the patients.

### 2.3. Criteria for positive diagnosis of brucellosis

(a) History of association with animals, with or without symptoms like fever, joint pains, chills, body ache and (b) detection of anti-*Brucella* antibodies by at least 2 serological tests [29] in significant titers (≥1:160 in case of STAT) [31].

### 2.4. Methods

Serum was separated from blood samples by centrifuging at 3000 rpm for 5 minutes. The samples were stored at 4 °C until further testing. All serum samples were tested using three serological tests: the Rose Bengal plate test (RBPT), standard tube agglutination test (STAT), and enzyme-linked immunosorbent assay (ELISA).

#### 2.4.1. RBPT

The RBPT is a spot agglutination test. In this test, 30 µL of *B. abortus* S99 colored antigen and 30 µL of patient serum was taken on a clean glass slide and mixed well. The test was interpreted as negative when agglutination was absent. When agglutination was present, the test was interpreted as positive and rated from 1+ to 3+. This was according to the strength of the agglutination observed from 1–3 minutes [29]. For RBPT, *B. abortus* S99 colored antigen was procured from Indian Veterinary Research Institute (IVRI, Bareilly), Uttar Pradesh, India.

#### 2.4.2. STAT

For the standard tube agglutination test (STAT), two-fold serial dilutions of the serum samples were prepared from 1:20 to 1:320 according to the Weybridge technique. The highest dilution of the serum exhibiting mat formation was considered as end point titer. A titer of 1:160 and above was considered significant for human brucellosis [29],[31].

#### 2.4.3. ELISA

Indirect ELISA testing for anti-*Brucella* IgM was performed using a commercially available ELISA kit (NOVALISA, NOVATEC, Germany). The indirect ELISA method was used because of its high sensitivity. The test was performed and results were interpreted as per kit literature.

### 2.5. Ethical statement

The study was approved (approval number: 2022-AS3) by Institutional Ethics Committee, School of Tropical Medicine, Kolkata, India.

### 2.6. Statistical analysis

The data obtained in this study was analyzed by R version 4.3.2 by R Foundation for Statistical Computing.

## 3. Results

In the present study, a total of 669 sera samples were collected from animal handlers and tested using three serological tests (RBPT, STAT, ELISA) for *Brucella* antibodies. Of these, 106 (15.8%) were diagnosed with brucellosis according to the pre-determined criteria. Among the total number of samples collected, 312 (46.6%) were males and 357 (53.4%) were females. Among those who were seropositive, 53 were male and 53 were female (Table 1). It is interesting to note that 99.7% (310 out of 311) of artificial insemination workers (AI workers) were males.

**Table 1.** Number and percentage of seropositivity in males and females found in this study.

Sex	Total Samples Collected	Seropositivity (N %)
Males	312	53 (17%)
Females	357	53 (14.8%)

Most of the samples were collected from the age group of 31–40 years (n = 362) followed by 41–50 years (n = 153). The largest percentage of seropositivity was noted in the age group of 51–60 years (23.5%). In this study, 8.8 % seropositivity was found in the age group of 21–30, 14.9% in the age group of 31–40, and 20.3% in the age group of 41–50 (Table 2).

**Table 2.** Table showing percentage of seropositivity in different age groups of animal handlers.

Age group (in Years)	Total Samples Collected	Total Samples Seropositive (N %)
<20	3	0 (0%)
21-30	90	8 (8.8%)
31-40	362	54 (14.9%)
41-50	153	31 (20.3%)
51-60	51	12 (23.5%)
>60	10	1 (10%)

Regarding district-wise distribution, the largest number of samples were collected from patients from the district of Nadia (166), followed by North 24 Parganas (83), and Bankura (82) of West Bengal. Only one sample was collected from Jhargram and Medinipur district. Among these, the sample from Jhargram was positive and the sample from Medinipur was negative. Among the two samples collected from the Malda district, one was positive and two out of four samples from Paschim Burdwan were positive. Other districts of West Bengal, such as Purba Medinipur (47.6%), Murshidabad (42.4%), Purba Burdwan (26.4%), displayed higher percentages of seropositivity (Table 3).

**Table 3.** Total seropositivity found in different districts of West Bengal, India.

Districts of West Bengal	Total Samples Collected	Total seropositivity in each district
Bankura	82	13 (15.8%)
Birbhum	24	2 (8.3%)
Burdwan	4	0
Cooch Behar	4	1 (25%)
Hooghly	30	5 (16.6%)
Howrah	25	1 (4%)
Jhargram	1	1 (100%)
Malda	2	1 (50%)
Medinipur	1	0
Murshidabad	33	14 (42.4%)
North 24 Parganas	83	15 (18%)
Nadia	166	19 (11.4%)
Purba Burdwan	34	9 (26.4%)
Paschim Burdwan	4	2 (50%)
Paschim Medinipur	21	4 (19%)
Purba Medinipur	21	10 (47.6%)
Purulia	2	0
South 24 Parganas	32	4 (12.5%)
Uttar Dinajpur	38	2 (5%)
Unknown	62	3 (4.8%)

### 3.1. Analysis of serological tests

Among the total number of tests performed, RBPT showed positive results in 124 (18.5%) patients. Only RBPT showed a positive result for three (0.4%) of the patients. However, significantly high STAT titers were found in 104 (15.5%) patients. All three tests showed positive results in 91 (13.6%) patients, while two out of the three tests (RBPT and SAT) showed positive results in 13 (1.9%) patients (Table 4).

**Table 4.** Results of serological tests (RBPT, SAT, and ELISA).

RBPT	SAT	ELISA	N (%)
+	+	+	91 (13.6%)
+	+	-	13 (1.9%)
-	-	-	535 (79.9%)
+	1:80	-	17 (2.5%)
+	-	-	3 (0.4%)

The overall prevalence of seropositivity was found to be 15.8% (95% confidence interval 13.2 to 18.8). It was not significantly different from the previously reported prevalence of 11% by Shukla et al. [12] with a *p* value of 0.89. Seropositivity has no association with age (p = 0.22), sex (p = 0.45), type of animal handler (p = 0.32), or mode of exposure (p = 0.13).

## 4. Discussion

Brucellosis, which is one of the neglected zoonotic diseases with economic importance, is either misdiagnosed or underreported in many parts of the world. Brucellosis has a strong occupational association, with certain professions being more commonly affected by the disease [22]. The disease can lead to serious complications in affected patients with an important public health issue.

Even though the continent of Asia comprises 60% of the world's population with India forming 17%, there are lacunae of studies reporting human brucellosis [9],[32]. There are studies on the concurrent existence of human and animal brucellosis exploring the epidemiology of this disease in veterinary professionals [22],[33]–[35]. However, to the best of our knowledge, there is no study documenting human brucellosis due to possible accidental exposure to the S19 vaccine in the Indian subcontinent. The present study provides valuable insights into occupational brucellosis. A study by Pereira et al. [36] in Minas Gerais, Brazil gives a detailed insight into accidental exposure to S19 and RB51 vaccines. The study revealed that one-third of the interviewed professionals had been accidentally exposed to the vaccine [36].

In the present study, routine serological tests (RBPT, STAT, and ELISA) have been used for the diagnosis of brucellosis. Here, among 669 animal handlers, 106 (15.8%) were diagnosed with brucellosis. The overall prevalence of seropositivity of 15.8% (95% confidence interval 13.2 to 18.8) was not significantly different from the previously reported prevalence of 11% by Shukla et al. [12] with a *p* value of 0.89. Previously, a high prevalence of occupational brucellosis was found in animal handlers (16.12%) demonstrated by Shome et al. [29], and this was in accordance with our findings. It may be inferred that the lack of knowledge about brucellosis and protective measures among animal handlers increases the probability of infection. The seropositivity distribution observed in males and in females was 17% and 14.8%, respectively (Table 1). This was higher compared to the data reported by Shome et al. [29], where they found 7.45% of males showed seropositivity and none of the females showed seropositivity. In our study, most of the samples were collected from the age group of 31–40 years. However, the highest rate of seropositivity was noted in the age group of 51–60 years (23.5%). The seropositivity was found to be 8.8% in the age group of 21–30, 14.9% in the age group of 31–40, and 20.3% in the age group of 41–50 (Table 2). In this study, the percentage of seropositivity was higher in the age groups of 31–40 and 41–50, compared to the previous study by Shome et al. [29]. High brucellosis seroprevalences were observed in the age groups 21–30 (8.90%), 41–50 (7.85%), and 31–40 (6.75%) by Shome et al. [29]. Regarding districts-wise distribution, the largest numbers of samples were collected from the district of Nadia (166), followed by North 24 Parganas (83) and Bankura (82) of West Bengal. We found variation in the seropositivity rate among the different districts. Statistical analysis showed that seropositivity has no association with age (*p* = 0.22), sex (*p* = 0.45), type of animal handler (*p* = 0.32), or mode of exposure (*p* = 0.13).

Human brucellosis has been reported earlier among pyrexia of unknown origin (PUO) cases, animal handlers, veterinarians, and slaughterhouse workers in India in some hospital based surveillance studies and case reports [37]–[41]. Our study concurs with the findings of similar studies on accidental exposure to the S19 or RB51 vaccine, where more than half of the target population recalled needle stick injuries [23],[42]. Vaccine bottle opening, syringe capping and recapping, and poor infrastructure were all significant risk factors of acquiring the disease. A study by Proch et al. [24] in India found more occupational brucellosis in veterinary assistants than among veterinarians. In our study, we have solely documented the disease in a cohort of animal handlers with relevant exposures.

The slow growth of *Brucella* in primary cultures delays diagnosis. Therefore, serological tests play a major role in the routine diagnosis of brucellosis [27],[43]. This was evident in our study when the initial 54 blood cultures of symptomatic patients showed no growth. Most of the diagnostic methods currently used for human serological testing use as antigen, whole “smooth” *Brucella* cells, or bacterial extracts containing high concentrations of sLPS [25]. Serological tests have problems of false positivity and negativity [27],[44]. Therefore, our study reiterates the fact that a single diagnostic test cannot be used to arrive at a diagnosis of human brucellosis.

Post exposure antibiotic prophylaxis has been recommended for humans accidentally exposed to anti-*Brucella* vaccines [45]. Based on literature on adverse events linked with vaccination campaigns, it is recommended that those concerned with the administration of this vaccine should wear gloves and eye protection to minimize exposure [26]. In spite of several efforts, the true burden of endemic brucellosis in our subcontinent remains to be seen. As there were no positive growths found in the blood cultures in this study, it could not be concluded if *B. abortus* strain 19 (S19) was responsible for brucellosis in the animal handlers. Therefore, more studies of human and animal brucellosis across the country are needed to distinguish between the transmission of the disease as a zoonotic disease and its transmission by other routes.

## 5. Conclusion

Brucellosis is a neglected disease whose problems are underreported worldwide, particularly in South Asia and India. Accidental exposure to the live S19 *Brucella* vaccine poses a significant threat to animal keepers in the Indian subcontinent. This study showed a seropositivity rate of 15.8% among Indian animal workers, suggesting a lack of awareness and protective measures among them. Female handlers had a seropositivity rate of 14.8%, while the rate of seropositivity for males was 17%. The highest seropositivity was found in the 51- to 60-year-old age group (23.5%). Recommendations include increased awareness, surveillance, improved safety measures through animal handler training, and prioritization of robust diagnostic tests like RBPT both in animals and humans. Moreover, animal brucellosis eradication programs needed to be implemented in order to control human brucellosis since the transmission is zoonotic.
